# The ROOTS study: a 10-year review of findings on adolescent depression, and recommendations for future longitudinal research

**DOI:** 10.1007/s00127-015-1150-y

**Published:** 2015-12-08

**Authors:** Gemma Lewis, Peter B. Jones, Ian M. Goodyer

**Affiliations:** Department of Psychiatry, University of Cambridge, Douglas House, 18b Trumpington Road, Cambridge, CB2 8AH UK; Department of Psychiatry, Cambridge Biomedical Campus, University of Cambridge, Box 189, Cambridge, CB2 0QQ UK

**Keywords:** ROOTS, Depression, Adolescence, Longitudinal, Neuroscience

## Abstract

**Purpose:**

The purpose of this study is to review longitudinal findings on adolescent mental health from the ‘ROOTS study’, and provide directions and recommendations for future longitudinal research. To do this, we discuss relevant findings from the ROOTS study, and review its strengths and limitations.

**Methods:**

We examined all publications from the ROOTS study up to July 2015, selected those examining adolescent mental health, and classified them as investigating (a) childhood risk factors for adolescent depression, (b) genetic and cognitive vulnerability to depression in adolescence, (c) genetic markers, childhood adversities, and neuroendophenotypes, (d) morning cortisol and depression, (e) physical activity and depression symptoms, and (f) the underlying structure of mental health in adolescence. We reviewed the strengths and limitations of the ROOTS study, and how they feed into recommendations for future longitudinal research.

**Results:**

There was evidence supporting a putative hormonal biomarker for the emergence of depression in boys. Environmental pathways from child adversity to adolescent depression were confirmed in girls, partly accounted for by negative life events in early adolescence. The preceding role of automatic cognitive biases assessed using behavioural tasks was substantiated, with evidence for genetic susceptibility. Novel latent statistical models of child adversity, depression, anxiety, and psychotic experiences were produced, with concurrent and prospective validity. Our experiences conducting the ROOTS study resulted in a set of strengths, limitations, and recommendations for future longitudinal studies.

**Conclusions:**

The ROOTS study has advanced knowledge on the aetiology of adolescent depression by investigating environmental, genetic, hormonal, and neural risk factors. Findings provide a foundation for future research integrating cognitive neuroscience with epidemiology.

## Introduction

Many mental illnesses begin in childhood or adolescence, and early interventions are advocated to delay or prevent onset [1, 2]. However, there are no generally accepted methods for preventing any mental illness. Treatments can be clinically effective, but recurrence of common mental illnesses is high [3–5]. A basic principle of epidemiology is that treatment and prevention depends on knowledge of causes. This remains a challenge for mental health research, as does testing causal hypotheses. Depression, for example, is the second leading cause of disability worldwide, but little is known about its causes [6].

In the absence of experimental designs, causal inferences can be strengthened using large, longitudinal, population samples, and appropriate statistical methods. Advantages include temporal ordering of events, minimisation of selective inclusion of participants, and adjustment for potential confounders as they occur [7]. A limitation is, often, lack of biological and behavioural data which is costly and difficult to collect from large samples.

The ROOTS study is an ongoing longitudinal population study that aimed to address this limitation by incorporating genetic, hormonal, behavioural, and neural data. Adolescent depression was the primary interest, but data were collected on psychotic experiences, anxiety, conduct problems, educational achievement, substance abuse, self-harm, and physical health. In July 2015, 19 articles from the ROOTS study had been published. Of these, 13 focused on adolescent mental health, and all included depression as an outcome variable. In this article, we describe the ROOTS study 10 years since it began. We aim to provide an example of a longitudinal population study with the novel inclusion of genetic, hormonal, behavioural, and neural data. In doing so, we review findings on adolescent mental health: we focus on adolescent depression, and provide directions for future research. We conclude with the strengths and limitations of our design, and recommendations for conducting future longitudinal population studies of this kind.

## The ROOTS sample and study aims

**Fig. 1:**
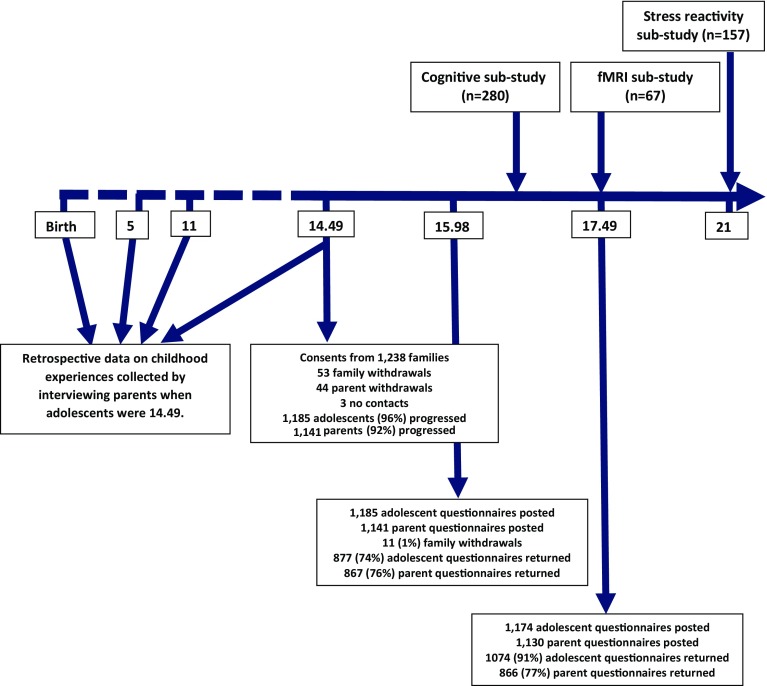
Timeline for the ROOTS study showing sub-studies and number of participants contacted and responding at each time point

The ROOTS study was funded by the Wellcome trust, and commenced fieldwork in April 2005. Recruitment was through secondary schools (*n* = 18) in Cambridgeshire, United Kingdom. 1238 adolescents were contacted (Fig. 1), 54.5 % female [8]. Three waves of data collection have taken place: when adolescents were an average age of 14.49, 15.98, and 17.49. These ages were chosen to span the period when incidence of depression begins to rise (puberty to late adolescence, peaking in the early to mid twenties [9]).

**Fig. 2:**
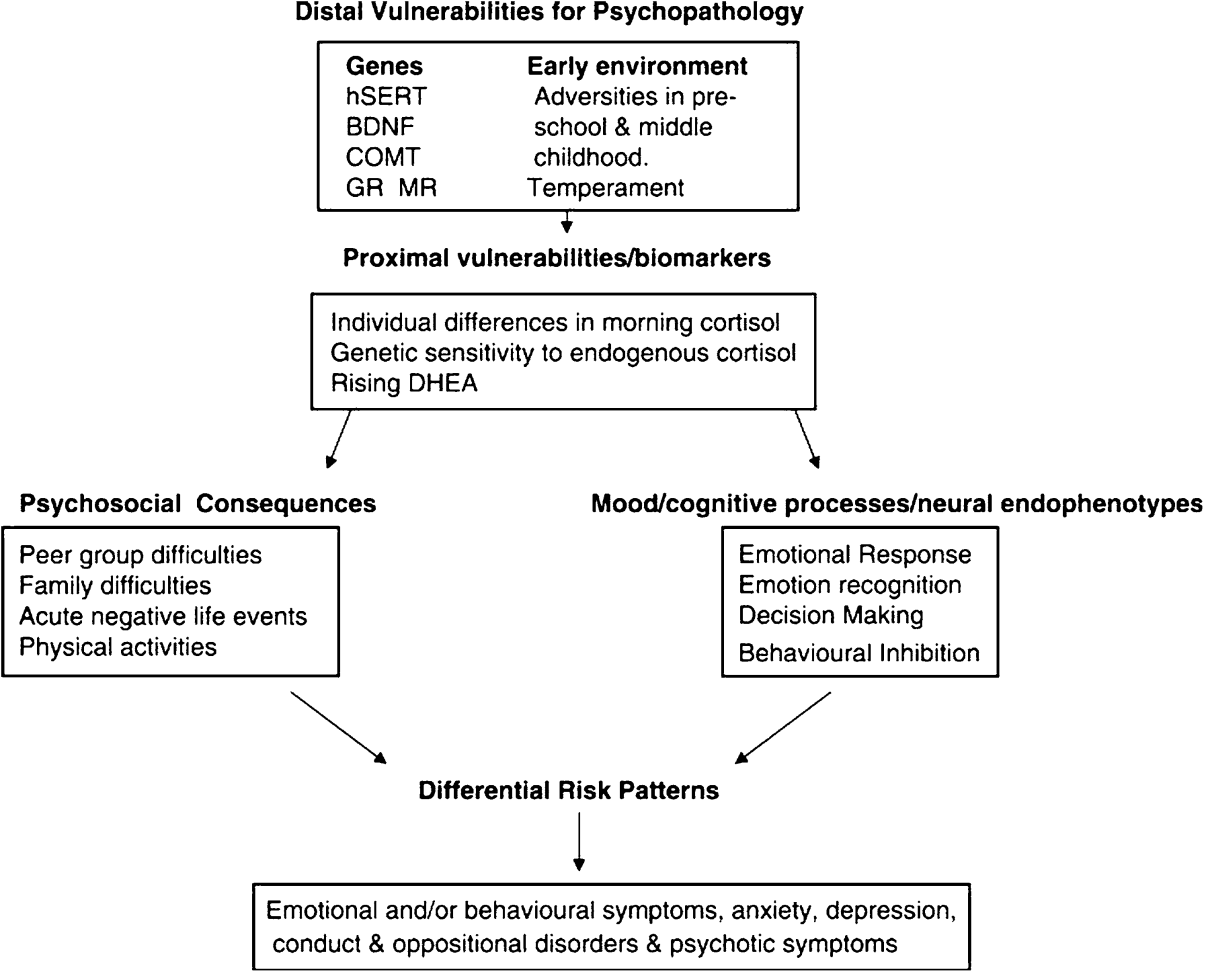
Theoretical model for the ROOTS study

The ROOTS study was based on a specific theoretical model (Fig. 2) [8]. The model followed evidence that adolescence is a time of biological (hormonal and neural) as well as psychological changes [10, 11]. For example, physiological changes such as increased gonadal hormones are hypothesised to affect organisation of brain circuitry [11]. Lack of biomarkers for mental illnesses impedes diagnosis and intervention, and is due, in large part, to limited understanding of biological factors. One way to access biology is through genetics [4]. The main aim of the ROOTS study was to discover genetically influenced intermediate biology (e.g. endophenotypes and biomarkers [12]) that preceded dimensional risk markers (e.g. sub-threshold symptoms) for future mental illnesses. Genetic and environmental associations were expected to vary at different ages, and identification of gender-specific pathways was emphasised. More recently, the aims of the ROOTS study aligned with the ‘research domain criteria’ (RDoc), proposed by the National Institute of Mental Health [13]. An advantage of designing a smaller longitudinal study, such as ROOTS, is that specific aims and hypotheses can be addressed in more detail. For example, the a priori aim to investigate adolescent depression meant that detailed data were collected specifically for this purpose.

## Measurement

At each time-point, self-reported questionnaire data were collected from adolescents, parents, and teachers (see [8] for list of instruments used). Depression symptoms were measured using adolescent reports on the Mood and Feelings Questionnaire (MFQ). Semi-structured diagnostic interviews were conducted with adolescents when they were 14 and 17. Depression was diagnosed using the Kiddie Schedule for Affective Disorders, present and lifetime version (K-SADS-PL). Saliva samples were collected at age 14, for genetic and hormonal data. We collected genetic and hormonal data based on previous findings implicating certain gene variants and hormones in depression aetiology (see [8] for a list of these data).

The ROOTS study adopted a longitudinal nested sub-study design. This was so specific hypotheses regarding cognition and the brain could be addressed cost-effectively. Three nested sub-studies have taken place (see Fig. 1). The cognitive sub-study focussed on frontal executive functioning. ‘Top-down’ (i.e. led by the cortex) processes of decision-making, behavioural inhibition, and reversal learning were assessed using the affective go-no-go, Cambridge gambling, and probability reversal learning tasks. Adolescents were selected based on their genotype for the serotonin transporter gene, and exposure to child adversity before the age of six. For the fMRI sub-study, participants were selected, again, on the presence or absence of child adversity, and their genotype for the serotonin transporter gene. The fMRI task used was previously shown to elicit amygdala activity [14]. Participants categorised the sex of 30 grey-scale photographs of angry, sad, and neutral faces (half female). The amygdala was selected as a region of interest based on evidence of its involvement in the processing of emotional information [15]. The stress reactivity sub-study assessed working memory at rest, and after exposure to a social stress test.

## Childhood risk factors for adolescent depression

As ROOTS was a study of adolescents, adversity in childhood was measured retrospectively at age 14 (see Fig. 1). Parents were asked to recall events early in their child’s life in a semi-structured interview: the Cambridge Early Experiences Interview (CAMEEI) [16]. As few retrospective studies had focussed on timing of adversity, data on three periods were collected: birth to age 5; ages 6–11; and ages 12–14 [16].

Measuring early family environments is not straightforward due to biases and inconsistencies in reporting [7]. The ROOTS study has made novel use of a latent variable statistical method to try to address this. CAMEEI data were analysed using latent class analysis (LCA). LCA assumes that responses to a set of variables indicate an unmeasured ‘hidden’ variable, with mutually exclusive classes or subtypes [17]. Identifying subtypes is of value because different subtypes have different characteristics. They may, therefore, have different prognoses and aetiologies. Traditional ways of grouping people, using cut-off scores on continuous measures, for example, are susceptible to measurement error. This is because continuous variables are a manifestation of the construct they intend to measure, so contain ‘nuisance variance’ [18]. We can, also, never determine where true cut-off points on continuous measures lie. They are arbitrary. Latent variables extract from questionnaire items only information that is relevant to the latent trait. They separate ‘construct’ from ‘nuisance’ variance, and reveal where natural groupings lie [18]. LCA can therefore be described as ‘person-centred’ rather than ‘variable-centred’ [17].

Four mutually exclusive subtypes of childhood adversity were identified in the ROOTS study [16]. The largest class (class 1) contained adolescents with a low probability of any adversity at any time-point (*n* = 784, 69 % of the sample). The second largest (class 2; *n* = 213, 19 % of the sample) had a higher probability (47 %) of family discord (e.g. marital disagreements). They also showed elevated rates of family loss, financial difficulties, and maternal psychiatric illness. The third largest (class 3; *n* = 76, 7 % of the sample) had a high probability (70–100 %) of inconsistent and atypical parenting by both parents (e.g. lax, very strict, cruel to be kind, smacking—all of which showed low prevalence). The smallest (class 4; *n* = 66, 6 % of the sample) had a high probability (>60 %) of physical and/or emotional abuse. Class membership was fairly stable (~55 %) over time, and complete escape from adversity by age 14 was uncommon: less than 25 % in the small but severe class, for example.

Of the four subtypes, three showed an ordinal relationship with psychiatric diagnoses at ages 14 and 17 [16]. Adolescents in the low adversity class (labelled ‘optimal’) had lowest risk of any psychiatric diagnosis (odds ratio 1.0). Risk of diagnoses increased in class 2 (‘labelled discordant’, odds ratio 2.3), and was highest in class 4 (labelled ‘hazardous’, odds ratio 4.0). The subtype characterised by inconsistent and atypical parenting (class 3; labelled ‘aberrant’) had weak associations with mental health outcomes so was comparable, in outcome, to the optimal subtype. Gender differences in mental health diagnoses at age 14 were strongest in the optimal class, with more females diagnosed (21 versus 9 % of males). In the discordant class, the gender difference was smaller (35 % females, 26 % males), and in the hazardous class, an approximately equal proportion of females and males were diagnosed (47 % females; 53 % males) [16].

One study tested whether distal influences of child adversity on risk for depression reduced over time, and found gender-differentiated pathways [19]. Discordant and hazardous subtypes were associated with increased adolescent depression symptoms in both genders, but the aberrant subtype only in girls. Across adolescence, associations between each subtype and depression symptoms decreased for boys, but remained for girls. Factors that ‘protect’ boys from depressogenic effects of child adversity warrant further investigation. One hypothesis is that there may be neurodevelopmental factors in adolescence that reduce depression symptoms in boys, but increase them in girls.

Identifying mechanisms of associations between child adversity and future psychiatric illnesses could inform early interventions. One ROOTS study found that, in girls, child adversity was associated with a higher number of negative life events by age 14 [19]. This association was not found in boys. Negative life events at age 14 were associated with depression symptoms at age 17 (adjusting for depression at age 14). This provides some evidence that associations between child adversity and depression in girls may be accounted for, in part, by continued exposure to adversity. This may include bullying by peers which is more likely in children exposed to family maltreatment, and is strongly associated with future depression [20, 21].

## Genetic and cognitive vulnerability to depression in adolescence

Only a proportion of children exposed to adversity become ill. This has led to investigations of environmental and individual factors that may increase (vulnerability) or decrease (resilience) propensity for mental illnesses. The ROOTS study examined moderation of child adversity by specific gene variants. Adversity before age 6 was associated with depression symptoms at age 14, only in adolescents with the short allele of the serotonin transporter gene [22]. This replicated a classic, but contested, finding of gene–environment interaction [23, 24]. There is some evidence that the serotonin transporter gene may increase susceptibility to environmental stress, by affecting brain function. Adolescents with a short allele of the serotonin transporter gene, who were exposed to child adversity, displayed an attentional bias to neutral and negative (but not positive) words on the affective go-no-go task. They were also worse at responding to neutral stimuli on the probabilistic reversal learning task, and made more errors when learning the task (prior to the reversal phase) [23]. In the sample overall, these cognitive parameters, measured at 16, were associated with diagnoses of depression and anxiety by age 17. Findings from the affective go-no-go task were consistent with a cognitive neuropsychological theory of depression: automatic biases towards negative, and away from positive stimuli, develop from early experiences, and play a causal role in depression [25]. Targeting automatic cognitive biases in treatments may lead to reductions in depression symptoms.

## Genetic markers, childhood adversities and neuroendophenotypes

There is evidence, from other studies, that child adversity, for example; maltreatment, is associated with changes to brain structure and function [26]. Brain regions proposed as sensitive to child adversity include the amygdala, prefrontal cortex, cerebellum, and visual cortices [26, 27]. These associations may involve structural differences such as variations in grey matter volume, and differences in reactivity to cognitive tasks. The ROOTS study aimed to address several methodological challenges to testing associations between child adversity and later neural outcomes. First, adjustment for events in-between child adversity and the neural outcome is required. These may include negative life events and psychiatric illnesses. For example, child adversity may have been associated with childhood psychiatric illnesses that affected brain development—rather than affecting brain development directly. Second, neural outcomes assessed in adolescence occur when the brain is undergoing structural changes. Separating effects of illness and adversity from age-related maturation is therefore required.

Two of the ROOTS studies have investigated associations between a genetic marker (the serotonin transporter gene), child adversity, and neural outcomes [22, 28]. First, an a priori region of interest strategy examined associations between the serotonin transporter gene, exposure to childhood adversities, and the amygdala, hippocampus and anterior cingulate cortex [22]. Second, a multivariate data-driven approach tested associations between correlated psychosocial variables and whole brain, with subsequent extraction of univariate associations; for example, with grey matter volume [28]. The psychosocial variables were: exposure to childhood adversities; proximal negative life events; psychiatric history; parental psychiatric history; adolescent self-reports of the quality of the family environment at age 14.49; and depression symptoms at age 14.49.

There was no evidence of an association between serotonin transporter genotype and grey matter volume, either as a main effect or in interaction with child adversity. However, main effect associations were detected for amygdala reactivity. Amygdala reactivity showed no evidence of an interactive association with the serotonin transporter gene and child adversity. These findings suggest that prior associations between amygdala reactivity and child adversity may have been confounded by genotype, recent life events, anxiety symptoms, or psychiatric history. This is important given evidence, in the ROOTS study, that child adversity is associated with increased negative life events in adolescents—for girls [19]. However, power to detect single gene effects in these studies was very low (*n* = 67). Low power can result in false positives [29].

As the ROOTS study could not adjust for ‘baseline’ neural measures, for example grey matter volume, conclusions are preliminary. This is akin to examining depression symptoms as an outcome, without adjusting for baseline levels: these may be individuals who had this level of grey matter volume at baseline. This is a general weakness of neuroscientific studies in psychiatry—longitudinal neural data are expensive to collect, and therefore rare. Inconsistent findings are also common, perhaps resulting from small sample sizes [29]. Replication in independent samples with repeated neural measures is required.

## Morning cortisol and depression

The ROOTS study has advanced knowledge of depression in boys by identifying a potential biomarker that was not indicative of depression symptoms in girls [30]. Using latent class analysis, adolescents were stratified into four groups based on ‘low’ or ‘high’ depression symptoms, and ‘low’ or ‘high’ cortisol (measured using morning saliva samples). Boys with high depression symptoms (over time), and high cortisol (at age 14), were at highest risk of depression diagnoses by age 17. They were more likely to have depression than boys with low depression symptoms and low cortisol, and boys with high depression symptoms and low cortisol. For girls, cortisol levels made no difference to the odds of depression diagnoses which were highest in those with high depression symptoms and low cortisol. Cortisol levels alone were not associated with depression in either sex. This is consistent with findings from the largest prospective study of cortisol and depression (*n* = 841), which found no evidence of an association, after adjustment for confounders [31]. Cortisol levels alone are unlikely to be pre-existing biomarkers in healthy individuals, but could mark depression as it emerges in boys. This could add to the predictive value of subclinical depression, and aid early detection in boys. It would be of value if future studies identified mechanisms that put boys with high cortisol and high depression symptoms at increased risk, but not girls. This could aid understanding of gender-differentiated neural and physiological pathways to depression. One hypothesis is that boys are more susceptible to potentially neurotoxic effects of raised cortisol.

## Physical activity and depression symptoms

Proximal risk factors for depression (e. g. sub-threshold depression symptoms and cortisol levels) can identify risk groups, but are difficult to modify. Proximal risk factors that could, more easily, be modified are of considerable interest. Physical activity is a priority for the public health agenda. Benefits for physical health are undoubted, and benefits for concurrent mental health are likely. However, evidence from the ROOTS study suggests that, in adolescence, levels of physical activity are not likely to be causally linked to future depression [32]. Unlike prior longitudinal studies using self-reported physical activity (subject to reporting biases), physical activity was measured objectively with heart rate and movement sensors. Physical activity at 14 was not associated with depression symptoms at 17, after adjustment for depression symptoms at 14, socioeconomic status, medication, pubertal status, and weight. There was also no cross-sectional association between physical activity and depression symptoms, contradicting prior findings [33]. Large randomised controlled trials are required to test whether physical activity is an effective treatment for clinically depressed adolescents. To our knowledge, this has not been done, except for one small pilot study [34]. Randomised controlled trials of the effects of exercise on depression have been conducted with depressed adults. There was some evidence of effectiveness, but studies were deemed too poor quality for a meta-analysis to reach a conclusion [35]. A relatively large, well-designed randomised controlled trial found that contact with a physical activity advisor increased physical activity, but did not reduce depression symptoms [36]. Evidence therefore supports the public health message that interventions to prevent and treat depression in adolescents, and adults, should not target physical activity.

## The underlying structure of mental health in adolescence

Comorbidity is the rule rather than the exception in psychiatry [37]. An underlying susceptibility to psychiatric illnesses could explain lack of causes, biomarkers, and treatments specific to individual illnesses [38].

**Fig. 3:**
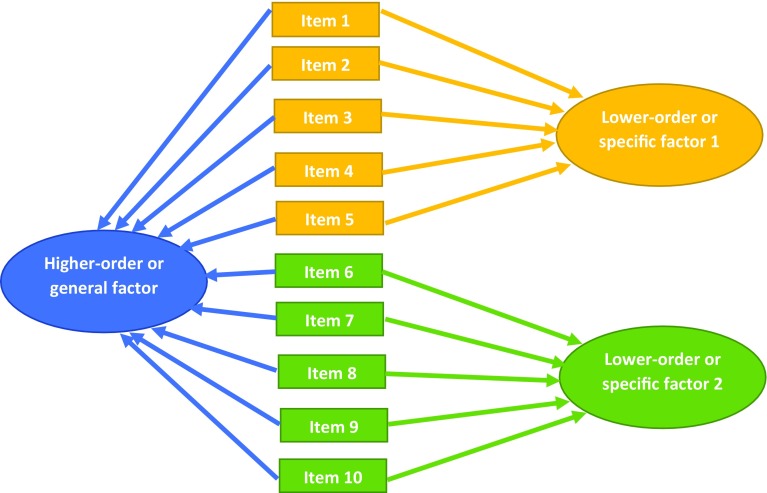
Diagram of the structure of a bi-factor model. All items load moderately to strongly on the general factor (*blue*), and subgroups of items load on the general factor and on one specific factor (*yellow* and *green*)

Several studies of the ROOTS cohort have developed statistical models to improve, methodologically, on existing outcome measures [39–42]. Two studies tested ‘bi-factor models’: the first using self-report measures of depression and anxiety [40]; the second, self-report measures of depression and psychotic experiences [41]. Bi-factor modelling, like latent class or factor analysis, creates latent variables [38, 40, 43]. In contrast, bi-factor modelling is described as ‘hierarchical’ because it produces a ‘higher-order’ latent variable that correlates, moderately or strongly, with all questionnaire items. The higher-order variable is often referred to as a general factor. ‘Lower-order’ factors account for residual variance not associated with the general factor. They normally only correlate with sub-sets of items (see Fig. 3). Genetic and neuroimaging research is suggestive of a common neurobiological basis for many psychiatric diagnoses [44, 45]. The utility of the bi-factor approach is, therefore, the potential identification of neurocognitive mechanisms that could ‘explain’ an underlying general susceptibility to psychiatric illnesses that is transdiagnostic.

For self-reported depression and anxiety symptoms at age 14, a general factor was confirmed, along with three specific factors. The general factor was labelled ‘general distress’, the specific factors: (1) hopelessness–suicidal ideation; (2) generalised worrying; and (3) restlessness–fatigue. Concurrent and prospective validity of the general distress bi-factor model was tested using psychiatric diagnoses at 14 and 17 [46]. Concurrently (at age 14), the general distress factor was associated with multiple diagnoses: depression, specific phobias, panic disorder, anxiety, eating disorders, substance abuse, conduct disorder and oppositional defiant disorder; but not ADHD or OCD. Prospectively, the general factor was associated with new onset depression and dysthymia, anxiety, and behaviour disorders. Prospective associations with other diagnoses were not explored due to lack of power. These findings are consistent with a general latent distress factor underpinning common anxiety, depressive, and behavioural disorders; but not impulsive or compulsive disorders.

**Fig. 4:**
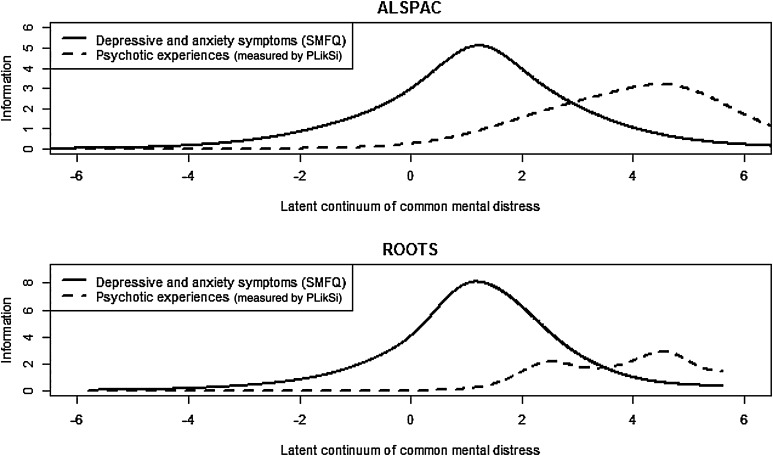
Information provided by items measuring depression symptoms and psychotic experiences on the latent continuum of common mental distress

One way of exploring, more precisely, ‘what’ general factors mean, is to examine where, along the distribution, items yield their maximum information. Psychotic experiences have low predictive value for schizophrenia [46]. They associate more strongly with concurrent than future depression symptoms [47]. There is also evidence that psychotic experiences mark increased severity of mental illness; they associate prospectively with suicidal thoughts and non-suicidal self-injury [48, 49]. These findings suggest that psychotic experiences and depression symptoms represent a common underlying illness, with psychotic experiences marking increased severity. These hypotheses were tested in ROOTS, and findings replicated in an independent longitudinal population sample: the Avon Longitudinal Study of Parents and Children (ALSPAC) [43]. Findings supported the existence of an underlying latent continuum for depression symptoms and psychotic experiences, represented by a general factor in a bi-factor model. Further analyses were conducted using an item-response-theory variant of factor analysis. This addressed the joint, or multivariate, distribution of items relating to depression symptoms and psychotic experiences, and tested measurement invariance—whether each item was measuring the same latent construct. Psychotic experiences were found to yield their maximum information at the severe end of this joint distribution. Depression symptoms provided most information at a lower point in the distribution (see Fig. 4). This argues against comorbidity as the relationship between depression and psychotic experiences. Rather, psychotic experiences may reflect the same underlying illness as depression symptoms, marking severity. In assessing only depression symptoms, the upper end of the latent trait, as it exists in the general population, may have been curtailed. This is akin to using a 25 centimetre ruler to measure a 30 cm problem—we may be missing important information. Diagnostic classification systems should, therefore, acknowledge that psychotic experiences are indicators of potentially severe depression, and increased risk of suicide attempt [43]. A common basis for depression and psychotic experiences has implications for understanding biology. For example, antidepressants can be clinically effective for psychotic depression, and antipsychotics for depressive episodes in patients with bipolar disorder. Pharmacological mechanisms may be relevant not for diagnoses, but for underlying neurobiology; indexed by a general mental illness trait. This is supported by a recent study identifying grey matter loss in the anterior insula and dorsolateral anterior cingulate cortex in patients with schizophrenia, depression, anxiety, bipolar disorder, addiction and obsessive–compulsive disorder. In healthy participants, these regions were found to form a neural network, and lower grey matter volume was associated with poorer executive functioning. A ‘transdiagnostic neural signature [44]’ supports findings from the ROOTS study suggesting that phenotypically linked illnesses have a common neural basis.

## Limitations and strengths of the ROOTS study; and recommendations for future longitudinal research

Participants from low socioeconomic statuses are under-represented in the ROOTS study. This is due to the demographic characteristics of Cambridgeshire relative to the rest of the United Kingdom. Findings should not, therefore, be generalised to more deprived neighbourhoods. A focus on adolescence meant that, unlike birth cohort studies, childhood experiences were reported retrospectively. This increased the likelihood of recall biases, and inconsistencies in reporting. Parents were sent a timeline in advance so they could consult records and photographs which may have gone some way to address this. Statistical power to study individual genetic effects in the ROOTS study was low, and longitudinal neuroimaging and behavioural data are lacking. Future sub-studies involving longitudinal neuroimaging and behavioural data are planned.

Despite high retention rates, attrition bias affects the ROOTS study. Adolescents who dropped out after Time one were more likely to have experienced child adversity (40.62 versus 27.40 %) and be of low socioeconomic status (21.22 versus 11.33 %). They also had higher depression symptoms [mean for participants who dropped out 17.32, mean for participants who were retained 14.81, *t*(1150) = −3.82, *p* = 0.0001]. Multiple imputation has been conducted to address this. Missing data from all three time points were imputed. Each imputation model contained all items from the measure being imputed (at each time point), and gender, socioeconomic status, and diagnoses at Time one (yes/no). Twenty imputations were created using the ‘ice’ command in Stata and Rubin’s rules for combining imputed datasets.

Challenges with recruitment were addressed in several ways. For example, initial responses to postal contact were low. When white paper was replaced with smaller, coloured cards; response rates improved markedly. Initial contact was made via post so the study had a presence in the home of participants. To encourage participation, the principal investigator and researchers gave presentations at year group assemblies in schools and colleges, accompanied by question and answer sessions. Contact with schools was maintained for 12 months after recruitment to support participation and maintain an active presence for the study.

Attrition was reduced through regular contact with participants, for example, hand-drawn birthday and Christmas cards. We also sent termly newsletters with updates on findings. As the study progressed, we contacted participants via text and email (common and convenient methods of communication for adolescents). One of the sub-studies also contacted participants via Facebook (specific ethical approval for this was required). The use of social media in longitudinal studies is recommended for creating a ‘study presence’ and disseminating findings. Lengthy and detailed retrospective interviews with parents meant that parents felt invested in the study, and encouraged adolescents to participate. For the cognitive, imaging, and stress sub-studies, participants were first selected (see “Measurement” section), then contacted and invited to participate. Cognitive and imaging studies took place on the research site in Cambridge, and participants were reimbursed for attending.

As part of the new ‘ROOTS 25’ project, we created a new website (http://www.roots.group.cam.ac.uk/). The website provides up-to-date information on findings, written in plain language. It also has a portal that participants can use to update their contact details. The website explains that the ROOTS study is joining the ‘Neuroscience in Psychiatry Network’ (NSPN: http://www.nspn.org.uk). NSPN is a collaboration between the University of Cambridge and University College London, and is studying development of the adolescent brain into early adulthood. By incorporating ROOTS participants, we will integrate their longitudinal information with up-to-date brain imaging and cognitive neuroscience studies. This provides an enrichment of existing longitudinal data (from childhood to adolescence). The ROOTS study will maintain its individual identity, with the same study logo.

By embedding behavioural and neuroimaging data in a longitudinal population sample, the ROOTS study addressed a preponderance of small, selective, case–control samples in cognitive neuroscience. This was a step towards uniting cognitive neuroscience and epidemiology—an important direction if the field is to understand complex interplay between environments, genes, physiology, and the brain.
